# Is the long-term poor prognosis of acute myocardial infarction in patients with mental illness mediated through their poor adherence with recommended healthcare?

**DOI:** 10.1093/eurpub/ckae005

**Published:** 2024-01-24

**Authors:** Giovanni Corrao, Matteo Monzio Compagnoni, Claudia Conflitti, Antonio Lora

**Affiliations:** Unit of Biostatistics, Epidemiology and Public Health, Department of Statistics and Quantitative Methods, University of Milano-Bicocca, Milan, Italy; National Centre for Healthcare Research and Pharmacoepidemiology, Department of Statistics and Quantitative Methods, University of Milano-Bicocca, Milan, Italy; Unit of Biostatistics, Epidemiology and Public Health, Department of Statistics and Quantitative Methods, University of Milano-Bicocca, Milan, Italy; National Centre for Healthcare Research and Pharmacoepidemiology, Department of Statistics and Quantitative Methods, University of Milano-Bicocca, Milan, Italy; Unit of Biostatistics, Epidemiology and Public Health, Department of Statistics and Quantitative Methods, University of Milano-Bicocca, Milan, Italy; National Centre for Healthcare Research and Pharmacoepidemiology, Department of Statistics and Quantitative Methods, University of Milano-Bicocca, Milan, Italy; Department of Mental Health and Addiction Services, ASST Lecco, Lecco, Italy; National Centre for Healthcare Research and Pharmacoepidemiology, Department of Statistics and Quantitative Methods, University of Milano-Bicocca, Milan, Italy; Department of Mental Health and Addiction Services, ASST Lecco, Lecco, Italy

## Abstract

**Background:**

Compared with patients without evidence of psychiatric symptoms, those with mental disorders experience reduced adherence with recommended healthcare and poorer clinical outcomes. This study aimed to evaluate whether the worse prognosis of patients with mental disorders after experiencing acute myocardial infarction could be fully or partially mediated by their reduced adherence to recommended healthcare.

**Methods:**

In this retrospective cohort population-based study, 103 389 residents in the Italian Lombardy Region who experienced acute myocardial infarction in 2007–19 were identified. Among them, 1549 patients with severe mental illness (SMI) were matched with five cohort members without evidence of mental disorders (references). Recommended healthcare (cardiac medicaments and selected outpatient services) was evaluated in the year after the date of index hospital discharge. The first occurrences of cardiovascular (CV) hospital admissions and any-cause-death were considered as endpoints. Mediation analysis was performed to investigate whether post-discharge use of recommended healthcare may be considered a mediator of the relationship between healthcare exposure and endpoints occurrence.

**Results:**

Compared with references, patients with SMI had lower adherence with recommended healthcare and adjusted risk excesses of 39% and 73% for CV hospitalizations and all-cause mortality. Mediation analysis showed that 4.1% and 11.3% of, respectively, CV hospitalizations and deaths occurred among psychiatric patients was mediated by their worse adherence to specific healthcare.

**Conclusion:**

The reduced use of recommended outpatient healthcare by patients with SMI had only a marginal effect on their worse prognosis. Other key factors mediating the prognostic gap between patients with and without mental disorders should be investigated.

## Introduction

Adherence with recommended healthcare for physical disorders has been reported to be poorer in patients living with severe mental illness (SMI) than in those without evidence of mental disorders.^1–3^ In addition, mental disorders have been associated with 2- or 3-fold increased risk of morbidity and mortality within six months after the occurrence of a cardiac event.^4^ Both these issues pertain to the so-called ‘physical healthcare gap’ of patients living with SMI.^1^^,^^5–7^ According with our best knowledge, this relationship has not yet been sufficiently clarified. That is, it is still unclear whether and to what extent the worse prognosis following a cardiac event among SMI patients may be explained: (i) by their limited adherence with recommended healthcare, for example due to the well-known mental health-related stigma hampering the care access and provision to patients affected by SMI,^8–10^ and/or (ii) by other factors,^11^ such as those related to the worse general physical conditions of SMI patients and to their unhealthy lifestyle behaviours.^12^

Given these premises, we carried out a real-world population-based cohort study aimed at clarifying the mechanisms underlying the physical healthcare gap among SMI patients. Specifically, as a novel contribution to the existing literature, we tested the hypothesis that the worse prognosis of patients with mental health disorders who experienced a myocardial infarction may be fully or partially mediated by their reduced adherence to recommended healthcare.

## Methods

### Data sources

The study was based on the computerized HealthCare Utilization (HCU) databases of Lombardy, a Region of northern Italy accounting for almost 10 million inhabitants (about 16% of the whole national population).

In Italy, all citizens have equal access to healthcare provided by the National Health Service (NHS), and an automated system of HCU databases is used to manage health services in each region, including Lombardy. HCU data include a variety of information on residents, such as diagnosis at discharge from public or private hospitals, outpatient drug prescriptions, specialist visits and diagnostic exams provided fully, or in part, free-of-charge by the NHS.

Furthermore, in Italy, from nearly 10 years a national automated information system on mental health care gathers data from the regional Departments of Mental Health (DMHs) accredited by the NHS. This information system (i.e. the so-called ‘Mental Health Information System’, MHIS) collects socio-demographic information, diagnostic and therapeutic codes for all patients receiving specialist mental healthcare by the regional DMHs’ facilities.^13^

These various types of data can be interconnected since a unique individual identification code is used by all databases for each NHS beneficiary. To preserve privacy, each identification code is automatically anonymized, the inverse process being only allowed to the Regional Authority upon request of judicial authorities. Further details on HCU database in the field of mental healthcare have been reported elsewhere.^13–15^ Diagnostic and drug therapy codes used for drawing records and fields from the considered databases are reported in the Supplementary table S1.

### Target population and cohorts’ selection

The target population consisted of all NHS beneficiaries’ residents in Lombardy aged 18 years or older between 2007 and 2019 (about 8.4 million inhabitants in 2019, http://demo.istat.it/index.html, last accessed 22 May 2023). Of these, patients who experienced a hospital admission via emergency room with diagnosis of acute myocardial infarction during the period from 1 January 2007 to 30 September 2019 were identified. The dates of admission and discharge of the first hospitalization occurred during the considered period were recorded as ‘index hospital admission’ and ‘index hospital discharge’, respectively. Transfers between wards, and even between hospitals, were considered as belonging to the same hospital stay. According to Supplementary figure S1, which reports a description of the cohort selection process, patients were excluded whether they: (i) were beneficiaries of the Regional Health Service (RHS) from less than 3 years before the index hospital admission; (ii) experienced at least a hospitalization for any cardiovascular event or disease within the 3 years before the index hospital admission; (iii) died for any cause during the index hospitalization; (iv) experienced an index hospitalization with a length of stay shorter than 3 days^16^ and (v) received at least three consecutive prescriptions for at least one class of cardiac drugs (i.e. anti-hypertensives, antiarrhythmics, statins, antiplatelets, beta-blockers, anticoagulants, statins) within the 6 months before the index hospital admission (in order to exclude patients with any previous cardiac treatment). The remaining patients constituted the first study cohort (hereafter referred to as ‘Step-1 cohort’). Among Step-1 cohort members, those who had been recorded in the MHIS for selected SMI (i.e. depressive, schizophrenic, bipolar or personality disorder) at any time prior the index hospital admission were identified. Diagnoses of SMI were considered as mutually exclusive, and patients who had more were classified to the most invalidating diagnostic group, referring to the following hierarchical classification: schizophrenic, bipolar, depressive and personality disorder.^17^

For each Step-1 cohort member affected by SMI, up to five controls were randomly selected from the remaining Step-1 cohort members without any evidence of mental disorders, to be matched for sex, age (± 3 years), date (± 30 days) and length (± 5 days) of the index hospital admission. The resulting risk sets (each including one SMI patient and the corresponding up to five reference patients), as a whole, formed the so-called ‘Cohort A’.

According to the user-only paradigm,^18^ patients belonging to Step-1 cohort without any dispensation of cardiac medicaments (see below) within one year after the index hospital discharge were also excluded, thus reducing the potential for confounding by indication. The remaining patients formed the second study cohort, hereafter referred to as ‘Step-2 cohort’. Again, SMI patients were identified, and 1:5 matching design was adopted to select reference patients without mental disorders by using the same criteria already described for the cohort A selection, thus identifying the ‘Cohort B’.

### Covariates

Baseline characteristics of cohort members included gender, age, and selected cotreatments and comorbidities, as detected from drug dispensations and hospital diagnoses within the three years before the index hospital admission.

Drug therapies included antidepressants, antipsychotics, mood stabilizers, antidiabetics, drugs for pulmonary diseases and antineoplastic agents. Comorbidities (e.g. diabetes mellitus, hypertension, etc.) were measured singularly, and through the so-called multisource comorbidity score (MCS). The MCS is a new comorbidity index derived from in-patient diagnostic information and outpatient drug prescriptions recently developed and validated in Italy,^19^ which was used for assessing the general clinical profile of each cohort member.

### Measuring post-discharge use of recommended healthcare

Out-of-hospital healthcare dispensed during the first year after the date of index hospital discharge was assessed. Seven classes of cardiac medicaments (renin–angiotensin system [RAS] blockade agents, beta-blockers, other blood pressure-lowering drugs, statins and other lipid-lowering drugs, antiplatelet, antiarrhythmic and anticoagulant agents) were considered.^20^ The period covered by each prescription was calculated according to the defined-daily-dose (DDD) metric. For overlapping prescriptions, the individual was assumed to have completed the previous one before starting the second one. Since we had no information about inpatient drug prescriptions, with the aim of avoiding the so-called immeasurable time bias,^21^ during each hospital stay cohort members were considered to be exposed to the same treatment as that recorded before the current hospital admission. Adherence with drug therapy was assessed through the ‘proportion of days covered’ (PDC),^22^ a quantity expressed by the cumulative number of days during which the medication was available, divided by the number of days of follow-up (usually 365). A patient was classified as exposed to drug therapy whether during the first year after the index hospital discharge (i) the drug was dispensed at least once (cohort A), or (ii) the drug was dispensed with a PDC ≥ 75% (cohort B).

As far as recommended outpatient services (i.e. cardiologic visit, echocardiogram, electrocardiogram [ECG], lipid profile testing and a cardiac rehabilitation programme), each cohort A member was classified as adherent whether, during the first year after the index hospital discharge, the service was dispensed at least once. Overall, a high adherence to recommended outpatient services was assumed to be reached for those patients who underwent all, or almost all, the recommendations (i.e. whether at least four of the five services were dispensed during the year).

### Follow-up and endpoints

The first occurrence of hospital admissions with diagnosis of any cardiovascular (CV) event or disease (please see Supplementary table S1), hereafter referred to as ‘CV hospitalization’, and death for any cause were separately considered as endpoints of interest. Starting from one year after the date of (i) the index discharge (cohort A) or (ii) the first dispensed drug (cohort B), cohort members accumulated person-time of observation until censoring (i.e. the earliest among death, CV hospitalization, migration or 30 September 2020).

## Data analysis

Patients with and without evidence of mental illness were compared for the baseline characteristics and the standardized mean difference (SMD) was calculated. For SMD, a cut-off of 10% or higher was used to identify meaningful differences between patients with and without SMI.^23^

The cumulative proportion of patients with and without evidence of SMI experiencing the considered endpoints was compared with the Kaplan–Maier estimator and the log-rank test. Cox proportional hazard model was fitted for estimating the hazard ratios (HR), and 95% confidence intervals (CI), for the association between severe mental illness and the considered endpoints. Adjustments for the above-listed covariates were made (please see the ‘Covariates’ subsection), and the proportional hazard assumption for the time-fixed covariates was tested by means of Schoenfeld residuals.

Logistic regression, or log-binomial regression where suitable,^24^ was used for estimating the odds ratios (OR), or risk ratios (RR), and 95% CI, for the association between severe mental illness and post-discharge recommended healthcare, after adjustment for the above-listed covariates.

Finally, mediation analysis^25^ was performed to assess whether post-discharge use of recommended healthcare may be considered a mediator of the relationship between mental health status (exposure) and endpoint occurrence (outcome). Indeed, mediation analysis investigates the mechanisms underlying an observed exposure–outcome relationship and examines how they relate to a third intermediate variable, the mediator.^26^ Thus, the total effect of the exposure on the outcome is decomposed into a direct and an indirect effect through the mediator variable.^25–27^ A Cox regression was used for measuring both *exposure → outcome* and *mediator → outcome* associations, while the *exposure → mediator* relationship was assessed by using a logistic regression, with the above reported additional information being used as covariates to control for confounding. Then, through methods for effect decomposition for mediation, the natural direct and indirect *exposure → outcome* association were estimated.^26^^,^^28^ The proportion mediated was also assessed, indicating how much of the whole outcome excess in SMI patients can be explained by the indirect effect of the mediator, in which the mental health status drives a change in the healthcare use and this change then affects the likelihood of experiencing the clinical outcome. Thus, the average causal mediation effect, which expressed the independent hazard (relative risk) associated with this indirect path, was calculated.

### Sensitivity analyses

All the main analyses have been stratified according with sex and individual categories of severe mental illness.

To overcome the arbitrary nature of the threshold used to define high adherence to recommended outpatient services, in secondary analyses, a more permissive definition of high adherence to outpatient services (at least three of the five services should be dispensed) was used.

Furthermore, to minimize the confounding effect of comparing two non-randomized groups of patients, data were also analysed according to a high-dimensional propensity score (HDPS) matching design.^29^^,^^30^ Therefore, the matching procedures for both cohorts A and B were repeated adding the HDPS (± 0.10) as a further matching variable, obtaining the HDPS-matched cohorts A and B.

### High-dimensional propensity score

The HDPS was calculated by means of the above-mentioned baseline covariates with the addition of other covariates automatically selected from the information of drug prescriptions and hospitalizations during the 2 years preceding the index hospital admission.^29^^,^^30^ The HDPS algorithm empirically identified and prioritized covariates that were believed to be proxies for unmeasured confounders. The 200 most predictive covariates were selected and included in a logistic regression model to estimate the propensity score (to be affected by SMI). The estimated HDPS was then considered as an additional matching variable in the cohorts A and B selection process.

### Statistical software

Mediation analysis was performed by using the R software (version 2.10.1/2022, R Foundation for Statistical Computing, Vienna, Austria), with the R Package ‘regmedint’ for Causal Mediation Analysis;^31^^,^^32^ whereas, all the other analyses were conducted with the use of Statistical Analysis System software (SAS, version 9.4; SAS Institute, Cary, NC, USA).

## Results

### Patients

As reported in Supplementary figure S1, about 256 000 beneficiaries of the RHS experienced at least one hospital admission for myocardial infarction between 1 January 2007 and 30 September 2019. Of these, 103 389 met the inclusion criteria, 1549 of whom had evidence of SMI and were considered as candidate to the study cohort. The initial cohort A was formed by 1497 SMI patients and 7119 matched references (as the failure of 1:5 matching pertained 52 SMI patients, 3.4%, and 366 references, 0.49%).

Baseline characteristics of cohort members with and without evidence of SMI are compared in table 1. The mean age (SD) was around 61 (12) years, and men were about two-thirds. Depression was the most represented mental health disorder in our setting. At baseline, SMI patients had higher use of antidiabetics and drugs for pulmonary diseases and a worsened comorbidity status (MCS > 2) than references.

**Table 1:** Baseline characteristics for cohort A members affected by severe mental illness (SMI, study cohort) and matched patients without evidence of SMI (reference cohort)^a^. Italy, Lombardy Region, 2007–20

	Study cohort (*n* = 1497)	Reference cohort (*n* = 7119)	**SMD (%)** ^b^
Gender			
Men	979 (65.4%)	4737 (66.5%)	MV
Age (years)			
Mean (SD)	61.5 (12.1)	61.4 (12.0)	MV
18–50	292 (19.5%)	1384 (19.4%)	MV
51–60	447 (29.9%)	2204 (31.0%)	
61–70	405 (27.1%)	1841 (25.9%)	
>70	353 (23.5%)	1690 (23.7%)	
Mental disorder			
Bipolar disorder	149 (10.0%)	–	NA
Depressive disorder	823 (55.0%)	–	
Personality disorder	180 (12.0%)	–	
Schizophrenic disorder	345 (23.0%)	–	
Clinical status^c^			
Good	696 (46.5%)	4312 (60.6%)	0.35
Intermediate	371 (24.8%)	1662 (23.3%)	
Poor	430 (28.7%)	1145 (16.1%)	
Previous use of:			
Antidepressants	778 (52.0%)	821 (11.5%)	0.96
Antipsychotics	496 (33.1%)	91 (1.3%)	0.93
Mood stabilizers	211 (14.1%)	69 (1.0%)	0.51
Antidiabetics	240 (16.0%)	792 (11.1%)	0.14
Drugs for pulmonary diseases	330 (22.0%)	1223 (17.2%)	0.12
Antineoplastics	19 (1.3%)	65 (0.9%)	0.04

SMI, severe mental illness; SMD, standardized mean difference; MV, matching variable; SD, standard deviation; NA, not available.

a For each Step-1 cohort member affected by SMI, up to five controls were randomly selected from the remaining Step-1 cohort members without evidence of mental disorders to be matched for sex, age, date and length of the index hospital admission.

b Standardized mean differences < 0.10 were considered as negligible and not statistically significant.

c The clinical status was assessed by using the multisource comorbidity score (MCS), according to the hospital admissions and the drugs prescribed in the three-year period before the date of the index hospital admission. Three categories of clinical status were considered: good (MCS score = 0), intermediate (1 ≤ MCS score ≤ 2) and poor (MCS score > 2).

### SMI and post-discharge healthcare

**Figure 1:**
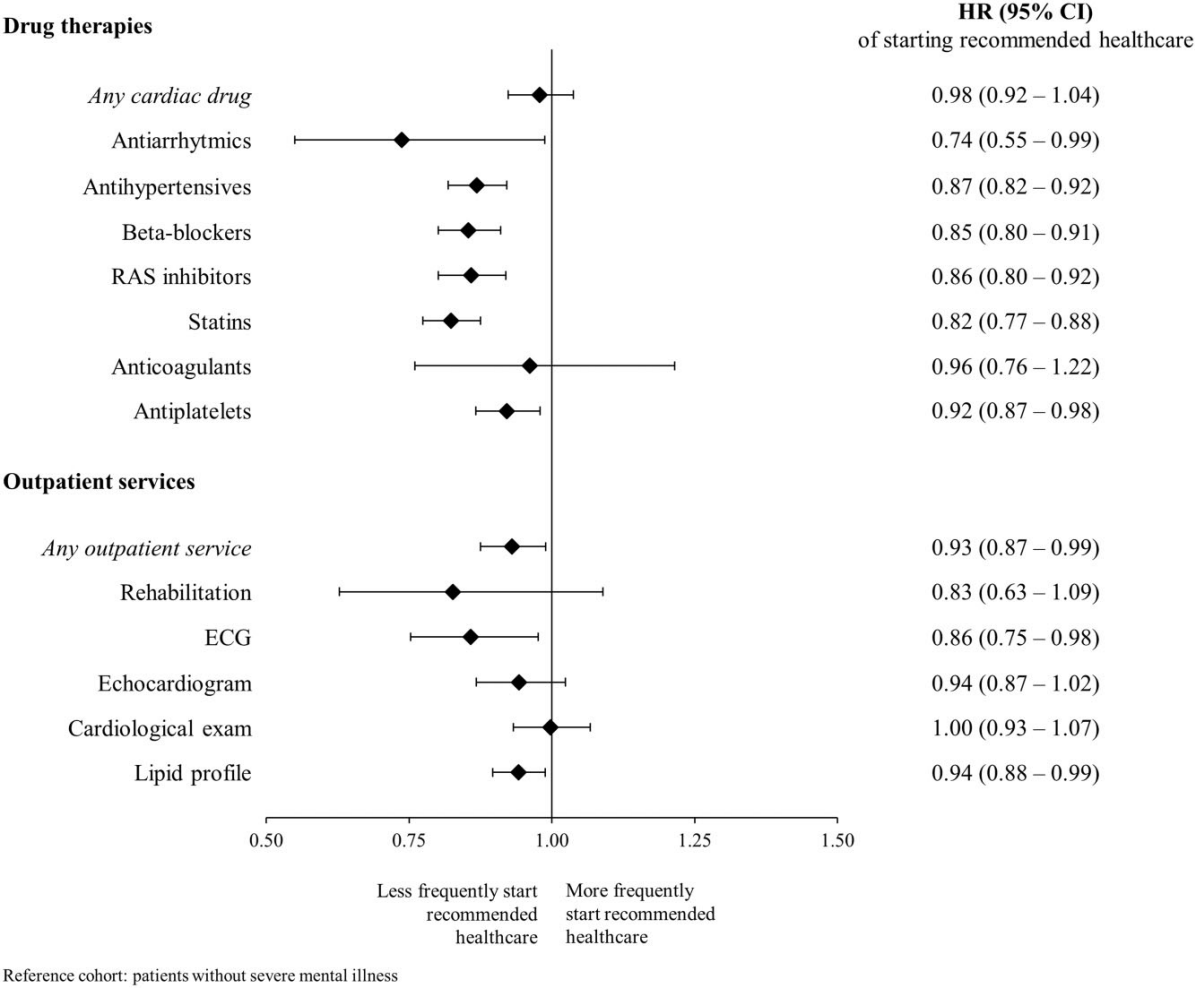
Effect of the presence of severe mental illness on the hazard ratio (HR) of starting recommended post-discharge healthcare, on cohort A members. Italy, Lombardy Region, 2007–20. Hazard ratio (HR) of recommended post-discharge healthcare, and 95% CI, was estimated according to the Cox proportional hazard model. Adjustments were made for covariates listed in table 1

Figure 1 shows that, during the year after the index discharge, compared with cohort A reference patients, those with SMI less frequently (i) started drug therapy with antiarrhythmics, antihypertensive agents as a whole, RAS blockade agents, beta-blockers, statins and antiplatelets, and (ii) experienced almost all the considered outpatient services, although only for ECG and lipid profile significance was reached.

Limited to patients who started drug therapy during the year after the index discharge (cohort B), those with evidence of SMI were less adherent with anti-hypertensives, RAS inhibitors, beta-blockers and statins than references (Supplementary table S2).

Results from sex-stratified analyses are displayed in Supplementary tables S3 and S4. Compared with references, patients with SMI were less likely to start drug therapies and were still less adherent with beta-blockers and statins for both males and females, whereas a lower adherence for anti-hypertensives and RAS inhibitors was observed only for males.

### SMI and post-discharge outcomes

**Figure 2:**
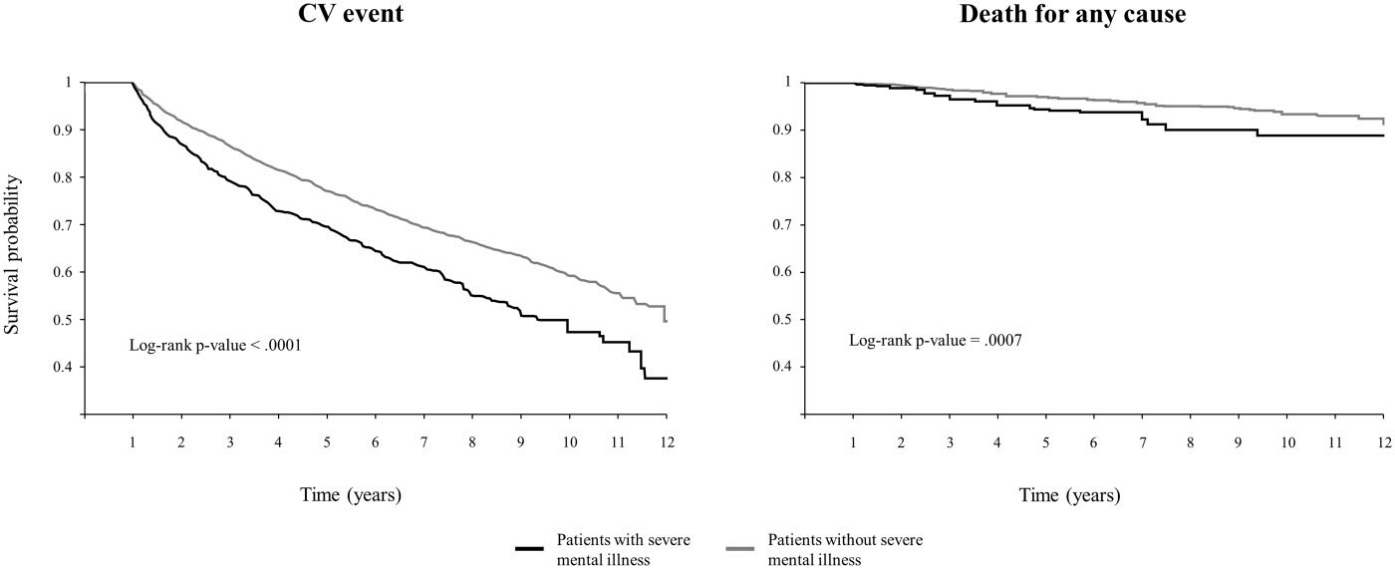
Cumulative incidence of cohort A patients experiencing hospital admission for any CV event or disease and/or death for any cause, according to the presence or absence of severe mental illness. Italy, Lombardy Region, 2007–20. The cumulative proportion of patients with and without evidence of severe mental illness experiencing the considered clinical outcomes was compared with the Kaplan–Maier estimator and the log-rank test

Overall, 32% of cohort A SMI patients, against 24% of reference cohort, experienced a further hospital admission for CV causes during follow-up. The corresponding figures for all-cause mortality were 4.7% and 3.0%. Cumulative proportions of CV hospitalizations and all-cause mortality were both significantly higher among SMI than reference patients (figure 2**)**, being the adjusted risk increased by 39% for CV hospitalizations (95% CI: 22–58%) and 73% for all-cause mortality (23–145%). Analyses stratified according with sex and individual categories of mental disorders are given in Supplementary tables S5 and S6 (Total Effect column).

### Mediation analysis

We decomposed the total effect of the *mental health status → outcome occurrence* association into natural direct and indirect effects mediated through post-discharge healthcare delivery (table 2). There was evidence of a natural effect, while the indirect one was not significant. The percentage of the excess risk of CV hospitalizations and all-cause deaths in SMI patients mediated through reduced adherence to recommended healthcare was 4.1% and 11.3%, respectively.

**Table 2:** Estimates of direct and indirect effects (mediated through the use of recommended post-discharge healthcare) of the association between the exposure to severe mental illness and the clinical outcome (CV hospitalization or death for any cause), on cohort B members. Italy, Lombardy Region, 2007–20

	**Natural direct effect HR_d_ (95% CI)** ^a^	**Natural indirect effect HR_i_ (95% CI)** ^a^	**Total effect HR_t_ (95% CI)** ^a^	**Proportion mediated (%)** ^b^
Clinical outcome				
CV hospitalization	1.36 (1.19; 1.55)	1.01 (0.98; 1.04)	1.37 (1.20; 1.57)	4.1
Death for any cause	1.64 (1.15; 2.33)	1.05 (0.97; 1.13)	1.72 (1.21; 2.45)	11.3

CV, cardiovascular; HR, hazard ratio; CI, confidence interval.

a Hazard ratios (HR) adjusted for the covariates listed in table 1.

b Outcome proportions mediated through high adherence to recommended post-discharge healthcare were estimated as follows: (HR_d_ * (HR_i_ *−* 1)/(HR_d_ * HR_i_ − 1)), where HR_d_ and HR_i_ refer to the corresponding Hazard Ratios for natural and indirect effect, respectively.

Sex-stratified analyses (Supplementary table S6), show a greater proportion mediated by reduced delivered healthcare for male patients, rather than females, for both outcomes.

### Sensitivity analyses

The main findings did not change substantially by modifying the threshold used to define adherence to outpatient services (Supplementary table S2, bottom part). Similar results were obtained for analyses performed on the HDPS-matched cohorts A and B. With respect to patients without mental disorders, those with SMI less frequently started any cardiac drug therapy (Supplementary table S7) and were still less adherent with anti-hypertensives, RAS inhibitors, statins, and outpatient services (Supplementary table S8), although for the latter significance was not reached. After having considered HDPS as an additional matching variable, the adjusted risk excess for all-cause mortality (64%; 95% CI: 3–162%) and CV hospitalization (26%; 5–51%) was similar to main analyses. HDPS-matched cohort B showed a similar proportion mediated for all-cause mortality, whereas considering CV hospitalization, the proportion mediated almost doubled (Supplementary table S9).

## Discussion

This large population-based investigation was based upon 100 000 patients who experienced a first myocardial infarction of whom nearly 1500 were on treatment for SMI. We found that, with respect to patients without evidence of SMI, those suffering from depressive, schizophrenic, bipolar and personality disorders had higher risk to be re-hospitalized for CV causes and to die, respectively, of 39% and 73% after the index hospital discharge, during a median follow-up of 4.7 years. According with a comprehensive meta-analysis,^33^ this finding supports the well-known notion that mental disorders worsen the prognosis of cardiac events. In the current study, we also found that during the first year after the index hospital discharge, SMI patients had a reduced use of recommended healthcare, including both drug therapy and outpatient services. These findings taken together, are consistent with several meta-analyses and primary studies emphasizing treatment gap and unmet needs in patients with mental disorders.^1^^,^^3^^,^^33^^,^^34^

According to several studies, a substantial proportion of mortality excess in psychiatric population can be considered potentially preventable through providing timely and high-quality specific healthcare.^11^^,^^35^^,^^36^ As a novel and original message, our study adds to these previous results the unexpected marginal role of the reduced access to post-discharge recommended healthcare of patients with SMI in explaining their worsened prognosis. Speculatively, this means that only a marginal reduction in the post-discharge morbidity and mortality risk is expected by improving the adherence with recommended healthcare. We suspect that the effect of several factors on worsening prognosis of SMI patients may be so large to obscure the effect of healthcare. Among these factors, the most relevant likely playing an important role are (i) the worsened general clinical profile^37^ (i.e. as also highlighted in table 1, with a 28.7% of SMI patients having a poor clinical status vs. the 16.1% of reference patients); (ii) lifestyle and behavioural patterns that lead to or exacerbate health problems^11^ (i.e. persons with SMI are more likely to smoke,^12^ suffer for alcohol and drug abuse, have a poor diet, experience inadequate physical activity and other unhealthy life habits, when compared with the general population^11^); (iii) the unawareness of SMI patients about their compromised physical status resulting in low motivation and treatment seeking^11^^,^^38^ and (iv) mental health-related stigma discouraging the provision of treatment and care to SMI patients.^8–10^

Therefore, a joint approach to physical and mental health should be promoted in public health policies.^11^ Specific interventions should be planned to overcome the stigmatizing attitudes and behaviours of healthcare professional towards mental health patients. Indeed, policies facing mental health disorder management and physical health treatment are needed, and should be integrated within a global multilevel framework, promoting interventions focused on individual (i.e. addressing lifestyle habits) and health-system (i.e. targeting health care providers) extents^11^ to reduce excess mortality.

## Strengths and limitations

The present study is unique in several respects. First, the investigation is based on data from a large, unselected population. Second, the availability of high-quality interconnectable individual data on outpatient and inpatient services supplied by the NHS offers the opportunity to trace the complete care pathway provided to patients. Thereby, the opportunity to generate reliable real-world evidence reflecting routine clinical practice, without being affected by selective participation or recall biases. Third, some methodological shrewdnesses, such as user-only design and the matching procedure, have been adopted for controlling confounding and other sources of bias (i.e. indication and/or immeasurable time biases). Furthermore, data were also analysed according to a HDPS matching design^29^ to address for both measured and unmeasured confounding, and, jointly with other sensitivity analyses, the robustness of the results was confirmed. Nevertheless, as an observational study, confounding cannot be ruled out, and future high-quality investigations are needed to confirm these findings.

Finally, limitations of this study should be considered to correctly portray our results. The first one is directly related to the data source. Indeed, the use of HCU databases did not allow to account for treatments dispensed in primary care and by private service providers (not accredited with the NHS), as well as out-of-pocket payments, representing a source of potential exposure and outcome misclassification. Despite that, it should be noted that patients taken-in-care by mental health services are likely expected to receive a more careful healthcare, making our results a potential underestimation of the real phenomenon, thus expected to be wider than estimated. Nonetheless, it should also be emphasized that HCU data are used to reimburse accredited and public healthcare providers and that incorrect and/or incomplete reporting leads to legal consequences, thus assuring on the high quality of the data source. As with any observational HCU data-based study, our investigation lacks relevant data, such as clinical severity, socioeconomic status and lifestyle habits. All these factors could potentially affect patients’ adherence and prognosis. However, all the results were adjusted for several cotreatments, comorbidities, for MCS (proxy of the patients’ clinical profile) and by HDPS matching, and we can reasonably exclude that worse outcomes in SMI patients might be due the presence of more or other comorbidities apart from CV disease or a worse clinical severity. Although the effect of some clinical factors and other unmeasured confounding has been controlled in our study by means of the matching procedure, user-only and HDPS-matched designs, further evidence evaluating the influence of social and clinical traits is needed. Lastly, by using HCU databases, the validity of the estimates on drug use is based on the assumption that the prescription of a drug corresponds to its consumption. This implies, however, that in the real-world, the actual use of drugs may be even still less than what observed.

## Conclusion

Through a mediation analysis on clinical outcomes experienced after myocardial infarction comparing patients with and without SMI, we showed that the reduced adherence to recommended healthcare experienced from patients with mental disorders had a marginal effect in explaining their worse prognosis. Thus, other key factors mediating the prognostic gap between patients with and without SMI should be implicated. Global approaches focusing not only on the physical treatments, but also considering the need for actions at individual and system-level should be recommended. Meanwhile, specific interventions should be planned to overcome the stigmatizing attitudes and behaviours of healthcare professional towards mental health patients.^3^^,^^10^^,^^39^^,^^40^

## Supplementary Material

ckae005_Supplementary_Data

## Data Availability

The data that support the findings of this study are available from the Region of Lombardy, but restrictions apply to the availability of these data, which were used under license for the current study, and so are not publicly available. Data are, however, available from the authors upon reasonable request and with permission of Lombardy Region. Key points Compared with patients without evidence of mental disorders, those living with SMI experience reduced adherence with recommended healthcare and poorer clinical outcomes. The reduced use of recommended outpatient healthcare from SMI patients had a marginal effect on their worse prognosis. Other key factors mediating the prognostic gap between patients with and without SMI should be implicated. Global approaches focusing not only on the physical treatments should be recommended. Specific interventions should be planned to overcome the stigmatizing attitudes and behaviours towards mental health patients.
