# Supplementary material for: Is the long-term poor prognosis of acute myocardial infarction in patients with mental illness mediated through their poor adherence with recommended healthcare?
Source: Eur J Public Health. 2024 Jan 24;34(3):584–91. doi: 10.1093/eurpub/ckae005 (PMC11161155; doi:10.1093/eurpub/ckae005)
Supplement: ckae005_Supplementary_Data [file ckae005_supplementary_data.zip › ckae005_Supplementary_Data/ejph-2023-06-om-0340-File006.pdf]

Is the long-term poor prognosis of acute myocardial infarction in patients with mental illness mediated through their poor adherence with recommended healthcare?

Running title: Prognosis after acute myocardial infarction in psychiatric patients

Giovanni CORRAO [1,2], ORCID id: 0000-0002-1034-8444, Matteo MONZIO COMPAGNONI [1,2], ORCID id: 0000-0002-2105-4572, Claudia CONFLITTI [1,2,3], ORCID id: 0009-0009-1502-4228, Antonio LORA [2,3], ORCID id: 0000-0002-6806-5199

[1] Unit of Biostatistics, Epidemiology and Public Health, Department of Statistics and Quantitative Methods, University of Milano-Bicocca, Milan, Italy
[2] National Centre for Healthcare Research and Pharmacoepidemiology, University of Milano-Bicocca, Milan, Italy
[3] Department of Mental Health and Addiction Services, ASST Lecco, Lecco, Italy

SUPPLEMENTARY MATERIAL

Address for correspondence: Dr. Matteo Monzio Compagnoni, PhD; Division of Biostatistics, Epidemiology and Public Health, Department of Statistics and Quantitative Methods, University of Milano-Bicocca, Street Bicocca degli Arcimboldi, 8, Building U7, 20126 Milan, Italy.

E-mail: matteo.monziocompagnoni@unimib.it

Supplementary Table S1. Diagnostic and therapeutic (ICD-9-CM, ICD-10, ATC and regional) codes used in the current study for drawing records and fields from Healthcare Utilization databases.

	Coding system	Codes
**Diseases**		
Cardiovascular event/disease	*ICD-9-CM*	390.* – 459.*
Acute coronary syndrome	*ICD-9-CM*	410.*, 411.0, 411.1, 412.*
Myocardial infarction	*ICD-9-CM*	410.*
Unstable angina	*ICD-9-CM*	411.0, 411.1
Old myocardial infarction	*ICD-9-CM*	412
Severe mental disorders		
Depression		
Depressive episode	*ICD-10*	F32.*
Recurrent depressive disorder	*ICD-10*	F33.*
Dysthymia	*ICD-10*	F34.1
Other persistent mood [affective] disorders	*ICD-10*	F34.8
Persistent mood [affective] disorder, unspecified	*ICD-10*	F34.9
Other recurrent mood [affective] disorders	*ICD-10*	F38.1
Other specified mood [affective] disorders	*ICD-10*	F38.8
Unspecified mood [affective] disorder	*ICD-10*	F39.*
Post-traumatic stress disorder	*ICD-10*	F43.1
Adjustment disorders	*ICD-10*	F43.2
Major depressive disorder, single episode	*ICD-9-CM*	296.2
Major depressive disorder, recurrent episode	*ICD-9-CM*	296.3
Atypical depressive disorder	*ICD-9-CM*	296.82
Unspecified episodic mood disorder	*ICD-9-CM*	296.90
Depressive type psychosis	*ICD-9-CM*	298.0
Dysthymic disorder	*ICD-9-CM*	300.4
Adjustment disorder with depressed mood	*ICD-9-CM*	309.0
Prolonged depressive reaction	*ICD-9-CM*	309.1
Depressive disorder	*ICD-9-CM*	311.*
Schizophrenic disorder		
Schizophrenia	*ICD-10*	F20.*
Schizotypal disorder	*ICD-10*	F21.*
Delusional disorders	*ICD-10*	F22.*
Brief psychotic disorder	*ICD-10*	F23.*
Shared psychotic disorder	*ICD-10*	F24.*
Schizoaffective disorders	*ICD-10*	F25.*
Other psychotic disorder not due to a substance or known physiological condition	*ICD-10*	F28.*

Unspecified psychosis not due to a substance or known physiological condition	*ICD-10*	F29.*
Schizophrenic disorders	*ICD-9-CM*	295.*
Delusional disorders	*ICD-9-CM*	297.*
Other nonorganic psychoses	*ICD-9-CM*	298.2, 298.3, 298.8, 298.9
Psychogenic paranoid psychosis	*ICD-9-CM*	298.4
Bipolar disorder		
Manic episode	*ICD-10*	F30.*
Bipolar affective disorder	*ICD-10*	F31.*
Cyclothymia	*ICD-10*	F34.0
Other single mood [affective] disorders	*ICD-10*	F38.0
Bipolar I disorder, single manic episode	*ICD-9-CM*	296.0
Manic disorder, recurrent episode	*ICD-9-CM*	296.1
Bipolar I disorder, most recent episode manic	*ICD-9-CM*	296.4
Bipolar I disorder, most recent depressed	*ICD-9-CM*	296.5
Bipolar I disorder, most recent episode mixed	*ICD-9-CM*	296.6
Bipolar I disorder, most recent episode unspecified	*ICD-9-CM*	296.7
Bipolar disorder, unspecified	*ICD-9-CM*	296.80
Atypical manic disorder	*ICD-9-CM*	296.81
Other	*ICD-9-CM*	296.89
Other specified episodic mood disorder	*ICD-9-CM*	296.99
Excitative type psychosis	*ICD-9-CM*	298.1
Personality disorder		
Specific personality disorders	*ICD-10*	F60.*
Mixed and other personality disorders	*ICD-10*	F61.*
Personality disorders	*ICD-9-CM*	301.*
**Drugs**		
Antiarrhythmics	*ATC*	C01B
Anticoagulant	*ATC*	B01AA, B01AE, B01AF
Antihypertensives	*ATC*	C02, C03, C07, C08, C09
Beta-blockers	*ATC*	C07
Renin-angiotensin system inhibitors	*ATC*	C09
Antiplatelet	*ATC*	B01AC04, B01AC05, B01AC06, B01AC22
Statins	*ATC*	C10AA
Antidiabetics	*ATC*	A10
Drugs for pulmonary diseases	*ATC*	R03
Antineoplastics	*ATC*	L01
Antidepressants	*ATC*	N06A
Antipsychotics	*ATC*	N05A, excluding N05AN

Mood stabilizers	*ATC*	N05AN, N03AX09, N03AG01, N03AF01
**Outpatient services**		
Cardiac rehabilitation	*Regional*	MAC06, MAC07, MAC08
Cardiological examination	*Regional*	89.01.3, 89.7A.3
Echocardiogram	*Regional*	88.72
Electrocardiogram (ECG)	*Regional*	89.50, 89.51, 89.44.1, 89.48.2
Lipid profile exam	*Regional*	90.13, 90.14.3, 90.43.2

Supplementary Figure S1. Flow-chart showing inclusion and exclusion criteria for the selection of the cohorts. Italy, Lombardy Region, 2007-2020.

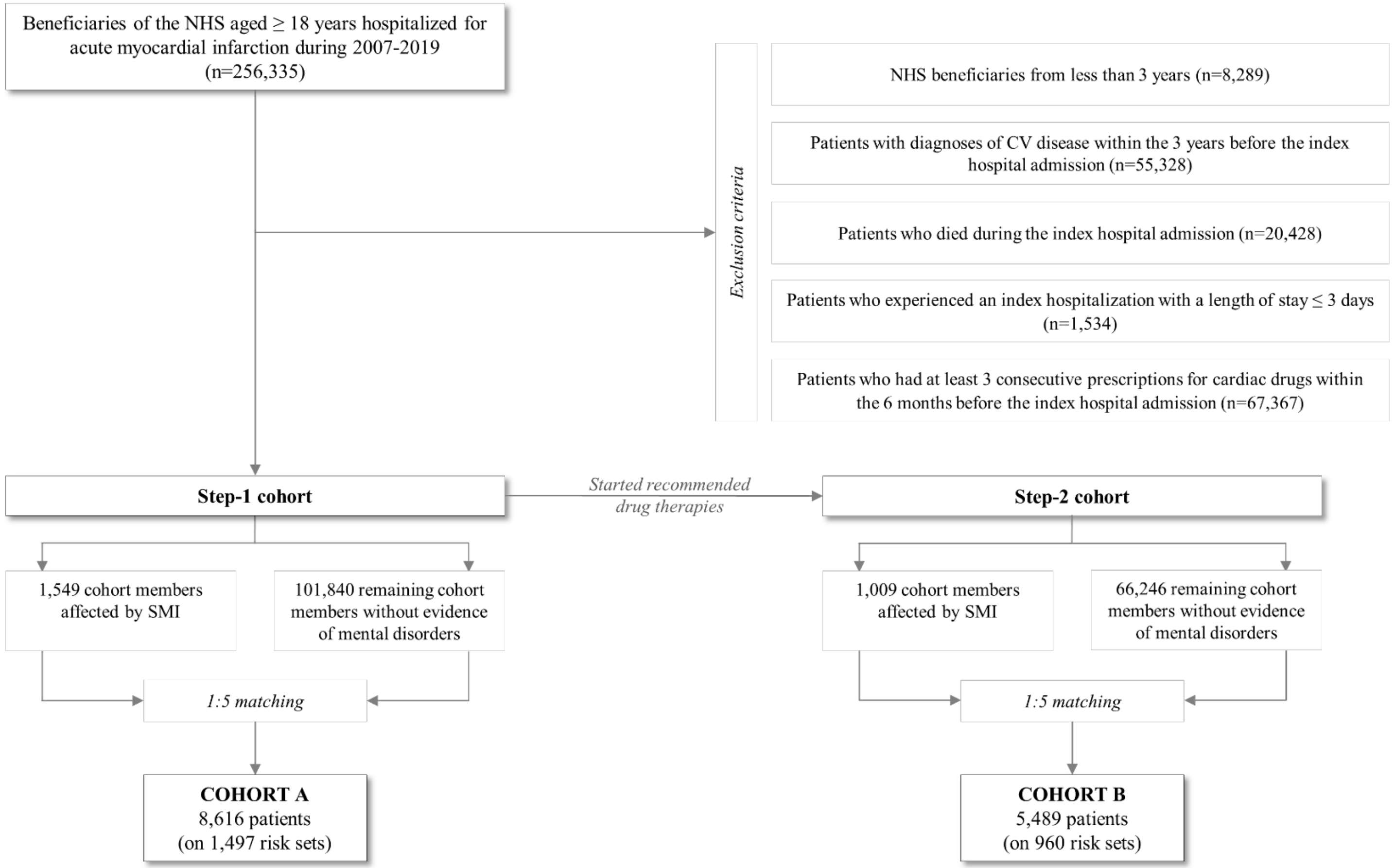

Footnote. For each step-1/step-2 cohort member affected by SMI, up to 5 controls were randomly selected from the remaining step-1/step-2 cohort members without evidence of mental disorders to be matched for sex, age, date and length of the index hospital admission. NHS, National Health Service; CV, cardiovascular; SMI, severe mental illness (depressive, schizophrenic, bipolar, and personality disorder).

Supplementary Table S2. Adjusted Odds Ratios (OR) or Risk Ratios (RR), and corresponding 95% CI, estimated for the association between exposure to severe mental illness and high adherence to recommended post-discharge drug therapies (cohort B) and outpatient services (cohort A). Italy, Lombardy Region, 2007-2020.

	Study cohort	Reference cohort	SMD (%)§	OR/RR (95% CI)¥
**Recommended drug therapies**				
Antiarrhytmics	9 (0.9%)	47 (1.0%)	0.01	0.91 (0.63; 1.31)*
Antihypertensives	459 (47.8%)	2458 (54.3%)	0.13	0.87 (0.82; 0.94)
Beta-Blockers	67 (7.0%)	449 (9.9%)	0.10	0.82 (0.72; 0.94)*
RAS inhibitors	324 (33.8%)	1898 (41.9%)	0.16	0.81 (0.74; 0.89)
Statins	641 (66.8%)	3393 (74.9%)	0.18	0.90 (0.86; 0.94)
Anticoagulants	7 (0.7%)	36 (0.8%)	0.01	0.93 (0.61; 1.40)*
Antiplatelets	593 (61.8%)	2811 (62.1%)	0.01	1.01 (0.96; 1.07)
**Recommended outpatient services**				
At least 4 out of 5 controls	91 (6.1%)	522 (7.3%)	0.05	0.83 (0.66; 1.05)*
At least 3 out of 5 controls	410 (27.4%)	2106 (29.6%)	0.09	0.93 (0.84; 1.04)
At least 1 out of 5 controls	5882 (82.6%)	1213 (81.0%)	0.14	0.93 (0.87; 0.99)

CI: Confidence Intervals; SMD: Standardized Mean Difference.
§ Standardized mean differences < 0.10 were considered as negligible and not statistically significant.
¥ Odds Ratios (OR), and Risk Ratios (RR), adjusted for the covariates listed in Table 1.
* Logistic regression model was used to estimate the association of interest.

Supplementary Table S3. Effect of the presence of severe mental illness on the Hazard Ratios (HR), and corresponding 95% CI, of starting recommended post-discharge healthcare, on cohort A members stratified according with sex. Italy, Lombardy Region, 2007-2020.

	HR (95% CI)¥	
	**Males**	**Females**
**Treatment initiation of…**		
Recommended drug therapies (1st prescription)		
Any cardiac drug	0.95 (0.89; 1.02)	1.04 (0.94; 1.15)
Antiarrhytmics	0.80 (0.55; 1.14)	0.66 (0.40; 1.09)
Antihypertensives	0.88 (0.82; 0.95)	0.84 (0.76; 0.93)
Beta-Blockers	0.85 (0.78; 0.91)	0.87 (0.78; 0.97)
RAS inhibitors	0.89 (0.82; 0.97)	0.80 (0.71; 0.90)
Statins	0.85 (0.79; 0.92)	0.77 (0.69; 0.86)
Anticoagulants	0.94 (0.70; 1.26)	1.01 (0.68; 1.50)
Antiplatelets	1.13 (0.89; 1.44)	0.92 (0.87; 0.98)
Recommended outpatient services (1st access)		
Any outpatient service	0.90 (0.84; 0.97)	0.98 (0.89; 1.09)
Rehabilitation	0.82 (0.59; 1.12)	0.85 (0.49; 1.49)
Ecg	0.87 (0.74; 1.02)	0.82 (0.66; 1.02)
Echocardiogram	0.98 (0.89; 1.08)	0.86 (0.74; 0.99)
Cardiological examination	1.01 (0.93; 1.10)	0.97 (0.87; 1.10)
Lipid profile	0.92 (0.85; 0.99)	0.98 (0.88; 1.10)

CI: Confidence Interval.
¥ Hazard Ratio (HR) adjusted for the covariates listed in Table 1.

Supplementary Table S4. Adjusted Odds Ratios (OR) or Risk Ratios (RR), and corresponding 95% CI, estimated for the association between exposure to severe mental illness and high adherence to recommended post-discharge drug therapies (cohort B) and outpatient services (cohort A), stratified according with sex. Italy, Lombardy Region, 2007-2020.

	OR/RR (95% CI)[¥]	
	**Males**	**Females**
**Recommended drug therapies**		
Antiarrhytmics	0.72 (0.45; 1.17)*	1.67 (0.87; 3.19)*
Antihypertensives	0.89 (0.82; 0.97)	0.84 (0.75; 0.95)
Beta-Blockers	0.81 (0.68; 0.95)*	0.84 (0.67; 1.06)*
RAS inhibitors	0.84 (0.79; 0.90)	0.73 (0.61; 0.87)
Statins	0.89 (0.85; 0.94)	0.93 (0.84; 1.02)
Anticoagulants	1.06 (0.67; 1.68)*	0.55 (0.19; 1.61)*
Antiplatelets	1.01 (0.93; 1.10)	1.01 (0.95; 1.08)
**Recommended outpatient services**		
At least 4 out of 5 controls	0.85 (0.64; 1.12)*	0.79 (0.52; 1.19)*

CI: Confidence Intervals.
¥ Odds Ratios (OR), and Risk Ratios (RR), adjusted for the covariates listed in Table 1.
***** Logistic regression model was used to estimate the association of interest.

Supplementary Table S5. Adjusted risk excesses, and corresponding 95% CI, for CV hospitalization and all-cause mortality in patients with severe mental illness, according with individual categories of mental disorders, on patients belonging to cohort A. Italy, Lombardy Region, 2007-2020.

	**Risk excess (95% CI)** ¥	
	**CV hospitalization**	**Death for any cause**
**Severe mental disorder**		
Schizophrenic	47% (13%; 92%)	161% (26%; 440%)
Bipolar	30% (-13%; 94%)	108% (74%; 481%)
Depressive	37% (15%; 64%)	68% (-16%; 238%)
Personality	23% (-16%; 79%)	-19% (-78%; 190%)

CI: Confidence Intervals; CV: Cardiovascular.
¥ The risk excesses were estimated as follows: *(HR-1)*100*; Hazard ratios (HR) for CV hospitalization and all-cause mortality were estimated using the Cox PH model and were adjusted for the covariates listed in Table 1.

Supplementary Table S6. Estimates of direct and indirect effects (mediated through the use of recommended post-discharge healthcare) of the association between the exposure to severe mental illness and the clinical outcome (CV hospitalization or death for any cause), on cohort B members stratified according with sex. Italy, Lombardy Region, 2007-2020.

Sex	Clinical outcome	Natural direct effect HR_d (95% CI)[¥]	Natural indirect effect HR_i (95% CI)[¥]	Total effect HR_t (95% CI)[¥]	Proportion mediated[§] %
Male	CV hospitalization	1.29 (1.10; 1.51)	1.04 (0.99; 1.10)	1.35 (0.85; 2.13)	16.3
	Death for any cause	1.58 (1.01; 2.46)	1.08 (0.96; 1.23)	1.71 (1.08; 2.71)	18.7
Female	CV hospitalization	1.45 (1.10; 1.90)	0.98 (0.95; 1.02)	1.42 (1.07; 1.88)	-6.8
	Death for any cause	1.85 (0.97; 3.54)	1.02 (0.95; 1.10)	1.89 (0.97; 3.67)	4.7

CV: Cardiovascular; HR: Hazard Ratio; CI: Confidence Interval.
¥ Hazard Ratios (HR) adjusted for the covariates listed in Table 1.
§ Outcome proportions mediated through high adherence to recommended post-discharge healthcare were estimated as follows: $(HR_d * (HR_i -1)/(HR_d * HR_i -1))$, where HR_d and HR_i refer to the corresponding Hazard Ratios for natural and indirect effect, respectively.

Supplementary Table S7. Effect of the presence of severe mental illness on the Hazard Ratios (HR), and corresponding 95% CI, of starting recommended post-discharge healthcare, on HDPS-matched cohort A members. Italy, Lombardy Region, 2007-2020.

Treatment initiation of…	HR (95% CI)[¥]
Recommended drug therapies (1st prescription)	
Any cardiac drug	0.99 (0.92; 1.07)
Antiarrhytmics	0.84 (0.59; 1.20)
Antihypertensives	0.85 (0.79; 0.91)
Beta-Blockers	0.79 (0.73; 0.86)
RAS inhibitors	0.87 (0.80; 0.95)
Statins	0.85 (0.78; 0.91)
Anticoagulants	0.87 (0.64; 1.18)
Antiplatelets	0.94 (0.87; 1.01)
Recommended outpatient services (1st access)	
Any outpatient service	0.91 (0.85; 0.99)
Rehabilitation	0.83 (0.59; 1.17)
Ecg	0.87 (0.74; 1.02)
Echocardiogram	0.94 (0.85; 1.04)
Cardiological examination	0.98 (0.90; 1.07)
Lipid profile	0.93 (0.86; 1.01)

CI: Confidence Interval; HDPS: High-Dimensional Propensity Score.
¥ Hazard Ratio (HR) adjusted for the covariates listed in Table 1. Estimates were obtained after having considered HDPS as an additional matching variable in the cohort selection process.

Supplementary Table S8. Adjusted Odds Ratios (OR) or Risk Ratios (RR), and corresponding 95% CI, estimated for the association between exposure to severe mental illness and high adherence to recommended post-discharge drug therapies (HDPS-matched cohort B) and outpatient services (HDPS-matched cohort A). Italy, Lombardy Region, 2007-2020.

**High adherence to…**	**OR/RR (95% CI)[¥]**
Recommended drug therapies	
Antiarrhytmics	1.03 (0.63; 1.70)*
Antihypertensives	0.92 (0.85; 0.99)
Beta-Blockers	0.90 (0.76; 1.07)*
RAS inhibitors	0.86 (0.77; 0.97)
Statins	0.89 (0.84; 0.94)
Anticoagulants	0.71 (0.41; 1.25)*
Antiplatelets	0.99 (0.92; 1.06)
Recommended outpatient services	
At least 4 out of 5 controls	0.85 (0.64; 1.12)

CI: Confidence Interval; HDPS: High-Dimensional Propensity Score.
¥ Odds Ratios (OR), and Risk Ratios (RR), adjusted for the covariates listed in Table 1. Estimates were obtained after having considered HDPS as an additional matching variable in the cohort selection process.
***** Logistic regression model was used to estimate the association of interest.

Supplementary Table S9. Estimates of direct and indirect effects (mediated through the use of recommended post-discharge healthcare) of the association between the exposure to severe mental illness and the clinical outcome (CV hospitalization or death for any cause), on HDPS-matched cohort B members. Italy, Lombardy Region, 2007-2020.

	**Natural direct effect** $\mathbf{HR_d}$ **(95% CI)**[¥]	**Natural indirect effect** $\mathbf{HR_i}$ **(95% CI)**[¥]	**Total effect** $\mathbf{HR_t}$ **(95% CI)**[¥]	**Proportion mediated**[§] **%**
**Clinical outcome**				
CV hospitalization	1.26 (1.05; 1.51)	1.02 (0.99; 1.06)	1.29 (1.07; 1.56)	10.4
Death for any cause	1.64 (1.03; 2.62)	1.05 (0.96; 1.15)	1.73 (1.07; 2.80)	11.5

CV: Cardiovascular; HDPS: High-Dimensional Propensity Score; HR: Hazard Ratio; CI: Confidence Interval.
¥ Hazard Ratios (HR) adjusted for the covariates listed in Table 1. Estimates were obtained after having considered HDPS as an additional matching variable in the cohort selection process.
§ Outcome proportions mediated through high adherence to recommended post-discharge healthcare were estimated as follows: $(HR_d * (HR_i -1)/(HR_d * HR_i -1))$, where HR_d and HR_i refer to the corresponding Hazard Ratios for natural and indirect effect, respectively.
