# Supplementary figures and images for: Is the long-term poor prognosis of acute myocardial infarction in patients with mental illness mediated through their poor adherence with recommended healthcare?
Source: Eur J Public Health. 2024 Jan 24;34(3):584–91. doi: 10.1093/eurpub/ckae005 (PMC11161155; doi:10.1093/eurpub/ckae005)

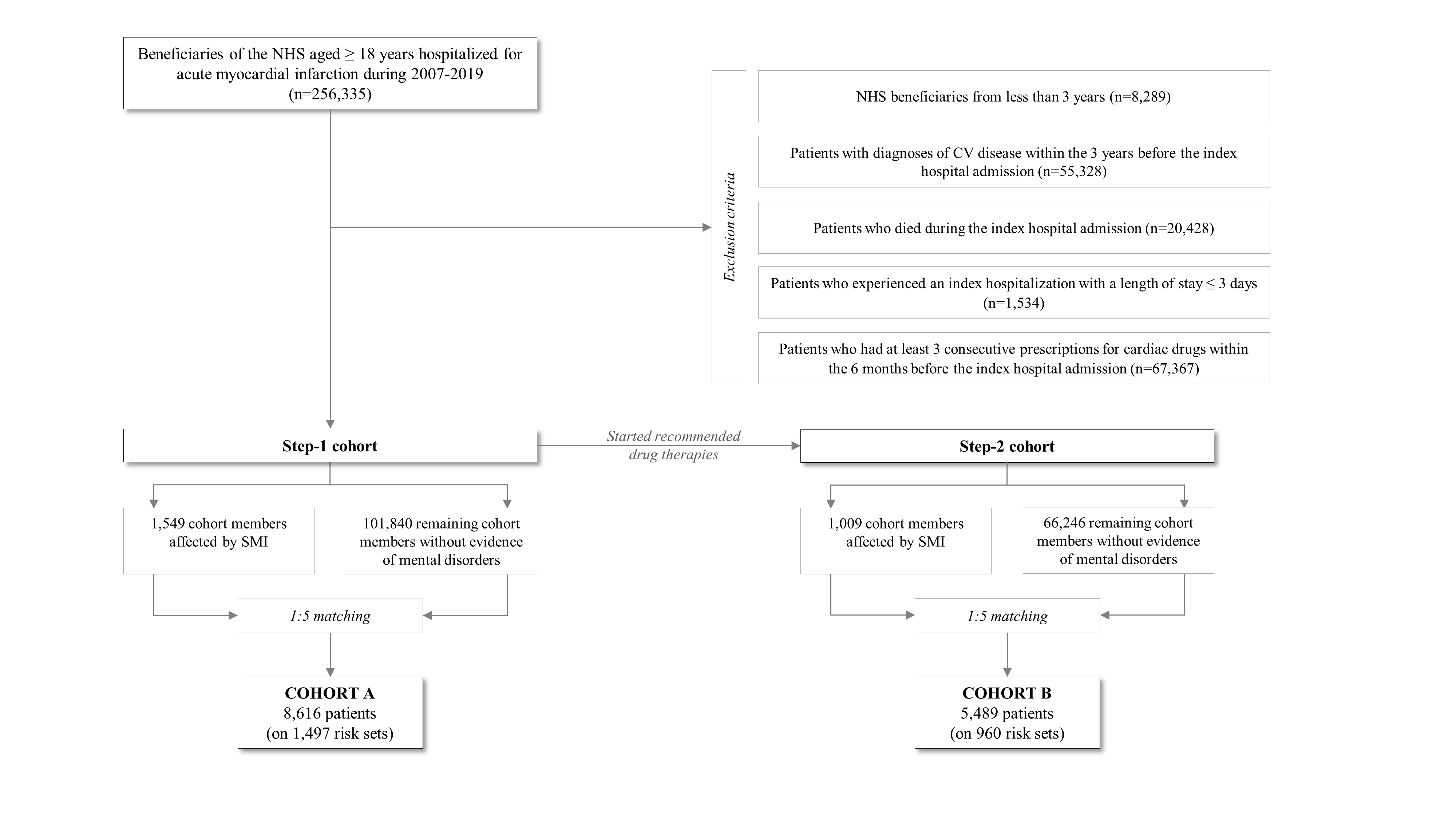

Supplement: ckae005_Supplementary_Data [file ckae005_supplementary_data.zip › ckae005_Supplementary_Data/ejph-2023-06-om-0340-File007.tif]
